# Microbial manipulators: *Fusobacterium nucleatum* modulates the tumor immune microenvironment in colorectal cancer

**DOI:** 10.1080/20002297.2025.2544169

**Published:** 2025-08-05

**Authors:** Qian Li, Wanyi Luo, Li Xiao, Xin Xu, Xian Peng, Lei Cheng, Xuedong Zhou, Xin Zheng

**Affiliations:** a State Key Laboratory of Oral Diseases & National Center for Stomatology & National Clinical Research Center for Oral Diseases, West China Hospital of Stomatology, Sichuan University, Chengdu, P.R. China; b Department of Cariology and Endodontics, West China Hospital of Stomatology, Sichuan University, Chengdu, P.R. China

**Keywords:** *Fusobacterium nucleatum*, colorectal cancer, tumor immune microenvironment, immune checkpoint blockade, microsatellite instability

## Abstract

*Fusobacterium nucleatum*, a microorganism ordinarily detected in the oral cavity, is considered as a pathobiont related to periodontitis and a range of human diseases, including colorectal cancer (CRC). The dynamics of how *F. nucleatum* encourages CRC tumorigenesis and progression has been well-investigated. Recently, mechanisms by which *F. nucleatum* regulates the tumor immune microenvironment (TiME) and subsequently alters CRC oncogenesis and advancement have drawn more and more attention. The TiME consists of immune cells and non-cellular components like cytokines in the tumor microenvironment. By contacting immune cells in the TiME, *F. nucleatum* fosters an immunosuppressive TiME, diminishes anti-tumor immunity and promotes CRC development. This also allows *F. nucleatum* to interfere with immunotherapy process and efficacy. In this review, we present a summary of how *F. nucleatum* interacts with immune cells within the TiME, thereby promoting CRC progression and influencing CRC immunotherapy effectiveness. This review also integrates insights from molecular pathological epidemiology (MPE) to contextualize host–microbe–environment interactions in CRC. We identify gaps in current knowledge and outline possible future research paths. These findings may offer valuable insights for future mechanistic research and the development of novel therapeutic strategies.

## Introduction

*Fusobacterium nucleatum*, a Gram-negative anaerobe prevalently exist in the oral environment, has been identified as a periodontal pathobiont [1–3]. It binds to various microorganisms through its adhesins such as RadD and acts as a bridging factor, leading to the co-gathering of microorganisms and biofilm formation, thus initiating periodontal disease [2,4]. Besides, host infection and irregular response prompted by *F. nucleatum* are also responsible for periodontal disease [2,4]. *F. nucleatum* has also been reported to be associated with other human diseases, including colorectal cancer (CRC) [4]. CRC stands as the third most prevalent cancer globally and the second most fatal. In 2022, there were 1,926,118 new cases and 903,859 deaths worldwide, accounting for 9.6% and 9.3% of all cancer cases, respectively [5]. The onset of CRC is affected by a blend of genetic and multiple environmental factors. Approximately 5–7% of CRC patients have hereditary colorectal cancer syndromes, with the majority being sporadic [6,7]. The incidence of CRC is significantly impacted by lifestyle factors such as smoking, diet, obesity, etc. and several studies have increasingly highlighted the contribution of the gut microbiota in tumorigenesis and progression of CRC [8–11]. *F. nucleatum* has been observed to be enriched in CRC tissues [12,13]. As an essential bio-marker for CRC diagnosis [14,15], *F. nucleatum* is associated with poorer prognosis [16], more severe pathological stage [17], and unfavorable treatment response [18–20].

Research on the mechanisms by which *F. nucleatum* promotes CRC development has been extensive. After transmitting through the digestive tract or circulation [15,21] from mouth to the intestines, *F. nucleatum* colonizes CRC tissues by fostering a suitable environment through the promotion of glycolysis in CRC cells [22] and by binding Fap2 to Gal-GalNAc on CRC cells [23]. Also, membrane fusion between *F. nucleatum* extracellular vesicles (FnEVs) and CRC cells plays a key role as it allows for the transfer of FomA, the outer membrane of FnEVs, and ensures it remains on CRC cells, which facilitates adhesion of *F. nucleatum* [24]. Additionally, *F. nucleatum* interacts with host colonic epithelial cells via surface molecules such as FadA and lipopolysaccharides (LPS), thereby boosting CRC proliferation [25–27]. *F. nucleatum* also facilitates CRC metastasis by upregulating Keratin7 [28] and stimulating EVADR/YBX1 pathway [29]. Clinical evidence and related studies indicate that *F. nucleatum* aids in chemotherapy resistance in CRC patients [18,20,30]. Beyond its established interactions with epithelial cells and tumor metabolism, recently, researchers have focused more on how *F. nucleatum* regulates the tumor immune microenvironment (TiME) and its impact on CRC advancement and immunotherapy outcomes. The tumor microenvironment (TME) includes a range of cells like inflammatory cells, carcinoma-associated fibroblasts, and various immune cells like lymphocytes, natural killer (NK) cells, macrophages, and neutrophils, along with non-cellular components produced by these cells [31]. The immune cells and cytokines in the TME, considered to be the main components of the TiME, interact with tumor cells and materially influence tumor progression and immunotherapy responses, presenting potential treatment targets [32–36]. Accumulated evidence indicates that *F. nucleatum* modulates the TiME towards anti-tumor immunity, driving CRC progression and affecting immunotherapy response. Here, we categorize the ramifications of *F. nucleatum* on various immune cells within the TiME based on traditional immune cell classification, and summarize current research on how modulation of the TiME by *F. nucleatum* promotes CRC progression and interferes with CRC immunotherapy responses, aiming to provide a reference for future mechanism studies and clinical practice in CRC prevention and treatment.

## Impact of *F. nucleatum* on the tumor immune microenvironment

*F. nucleatum* directly engages with immune cells, such as T lymphocytes, macrophages, neutrophils, and myeloid-derived suppressor cells (MDSCs). Through modulation of their density, effector functions, and polarization, it promotes immune suppression and disrupts antitumor responses, although under certain conditions, it may also contribute to enhanced antitumor immunity.

### Impact of F.nucleatum on lymphocytes

*F. nucleatum* has been shown to influence both the density and functionality of tumor-infiltrating lymphocytes (TILs) through direct interactions and immunomodulatory metabolites. These alterations contribute to immune evasion and disease progression, with some effects dependent on the tumor’s microsatellite instability (MSI) status.

#### Regulation of TIL density and subpopulation proportions by F.nucleatum

Numerous studies demonstrate that a high density of TILs is indicative of an improved prognosis for CRC, independent of other factors [37–39]. Cross-sectional studies by Mima et al. [40] on 598 CRC patients and Borowsky et al. [41] on 933 CRC patients both suggest an inverse relationship between the abundance of *F. nucleatum* and CD3^+^ T cell density in CRC tissues. Despite possible reverse causation due to the limitation of cross-sectional studies, by combining mechanism research [42–44] suggest *F. nucleatum* can attenuate T cell immune response which will be elaborated in the following sections and these cross-sectional studies, we can conclude that *F. nucleatum* promotes CRC progression by reducing TIL density.

However, another research by Hamada et al. indicates that the association of *F. nucleatum* with TIL infiltration in CRC depends on the microsatellite instability (MSI) status of the tumor. In MSI-high (MSI-H) CRC, a higher presence of *F. nucleatum* is associated with a lower density of CD3^+^ T cells in CRC tissues, whereas in non-MSI-H CRC, this association reverses [45]. Microsatellites (MS) are sequentially repeated short sequences dispersed across the human genome that are susceptible to DNA replication error [46]. Deficient DNA mismatch repair system (dMMR) causes the MSI while proficient mismatch repair system (pMMR) engenders the MSS [46]. Five markers, including mononucleotide loci and dinucleotide repeats, are taken into consideration to classify tumors by MSI, and if size alteration of at least two of the five markers is observed in tumors compared to normal tissue, they are classified as MSI-H, while others are defined as non-MSI-H tumors [47,48]. MSI status is directly related to the intratumoral immunological characteristics of CRC [49–51]. MSI-H CRC features a higher tumor mutational burden (TMB), a greater capacity to carry neoantigens, and an increased density of TILs, whereas non-MSI-H CRC is associated with TIL deficiency and an immunosuppressive TME. Additionally, *F. nucleatum* is correlated with MSI-H status in CRC [16]. The interaction among *F. nucleatum*, MSI status, and TILs suggests that the interplay of *F. nucleatum* and TIL density should be analyzed in conjunction with MSI status. Since about 15% of CRC are MSI-H [52], it turns out that according to Hamada et al., TILs are positively correlated with *F. nucleatum* in most cases, which is seemingly contradictory to the findings of Mima and Borowsky et al. Results of these cross-sectional studies may be affected by inadequate control of selection bias, sample size, whether to conduct stratification based on MSI status, unidentified confounding factors and variable category distinction, some of which are hard to avoid, calling for further research for a unified conclusion.

Considering the functional differences among T lymphocyte subsets, the correlation between *F. nucleatum* and the density of different T lymphocyte subsets has various implications. But overall, *F. nucleatum* regulates TILs towards a poorer prognosis for CRC. High densities of tumor-infiltrating CD8^+^ T cells indicate considerably lower CRC recurrence risk [38], while CD8^+^ T cells are significantly reduced in CRC tissues and liver metastasis specimens with *F. nucleatum* compared to those without [53,54]. Tumor-infiltrating CD45RO^+^ cells (memory T cells) are associated with longer survival in CRC patients [55], and Borowsky et al. found a negative correlation between *F. nucleatum* and CD45RO^+^ cell density in tumor stroma [41]. Kim et al. discovered that *F. nucleatum* is positively correlated with the density of exhausted CD8^+^ and FoxP3^+^ T cells, leading to an immunosuppressive TME [56]. Formate secreted by *F. nucleatum* contributes to a pro-inflammatory TME in favor of tumorigenesis [57]. These studies indicate that *F. nucleatum* not only regulates the number of TILs but also modulates the proportions of different TIL subsets to promote CRC progression.

Mechanisms underlying the association between *F. nucleatum* and TIL density are not fully elucidated. Succinate, a metabolite of *F. nucleatum*, can inhibit the migration of CD8^+^ T cells to tumor tissues by inhibiting cGAS-mediated IFN-β production, and downregulating Th1 chemokines such as CCL5 and CXCL10 [19]. *F. nucleatum* can induce lymphocyte death through its outer membrane proteins Fap2 and RadD [58].

#### F.nucleatum suppresses the immune response of TILs

**Figure 1.**
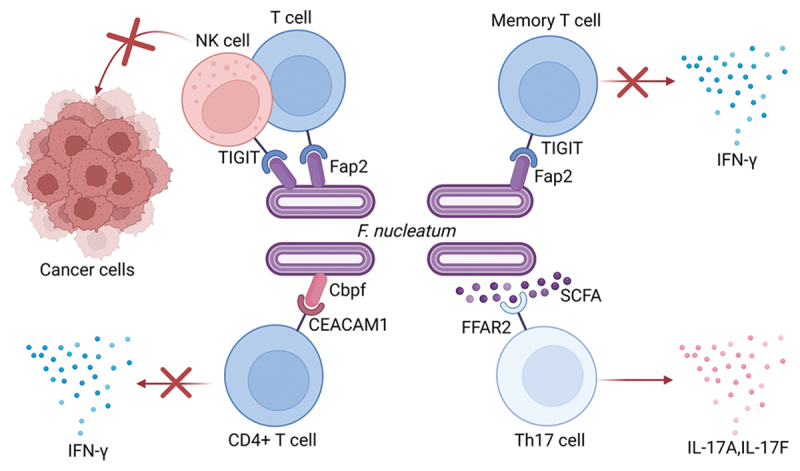
*F. nucleatum* suppresses the immune response of TILs through surface proteins and metabolites. The binding of Fap2 on *F. nucleatum* to TIGIT receptors on NK and T cells could attenuate their ability to attack tumor cells and IFN-γ secretion of memory T cells. CEACAM binding protein of Fusobacterium (CbpF) on the surface of *F. nucleatum* can bind to CEACAM1 on CD4^+^ T cells, hampering their immune response. Short-chain fatty acids (SCFA), metabolites of *F. nucleatum*, activates FFAR2 on Th17 cells, leading to pro-inflammatory secretion.

The mechanisms by which *F. nucleatum* modulates T cell immunity are summarized in Figure 1. Notably, *F. nucleatum* can inhibit lymphocyte function through surface proteins, promoting tumor growth. Fap2, adhesin of *F. nucleatum*, can attach to the TIGIT receptors on NK cells and TILs, transmitting inhibitory signals through the ITIM and ITF motifs in TIGIT’s cytoplasmic tail, thus inhibiting the cytokilling effect of NK cells and TILs. Fap2 also inhibits IFN-γ secretion by CD4^+^ memory T cells [44]. CEACAM binding protein of Fusobacterium (CbpF) on *F. nucleatum* can engage with the N-terminal domain of CEACAM1 on CD4^+^ T cells, suppressing their function [43]. Besides, FFAR2 on Th17 cells can identify short-chain fatty acids (SCFA), metabolites of *F. nucleatum*, thus stimulating Th17 cells to produce IL-17A, IL-17F, and creating a pro-inflammatory TME conducive to tumor growth [42]. In ESCC, *F. nucleatum* can stimulate high expression of KIR2DL on CD8^+^ T cells, resulting in immune suppression and tumor immune evasion [59]. *F. nucleatum* also notably inhibits the production of IFN-γ and TNF-α by CD8^+^ T cells in ESCC [60]. These findings raise the question of whether similar immune-modulatory effects occur in CRC. It is plausible that F. nucleatum may employ comparable mechanisms – such as KIR family receptor signaling or cytokine inhibition – to impair CD8^+^ T cell functionality in the CRC tumor immune microenvironment. If confirmed, this would suggest a conserved strategy by F. nucleatum across different gastrointestinal malignancies to suppress anti-tumor immunity.

#### F.nucleatum and the peritumoral lymphocytic reaction

The peritumoral lymphocytic reaction in CRC is associated with and reduced tumor invasiveness and more promising prognosis [37]. It has been reported that *F. nucleatum* exhibits an inverse relationship with the peritumoral lymphocytic reaction in esophageal cancer, suggesting its potential role in dampening local immune responses [61]. However, no study to date has definitively characterized the effect of *F. nucleatum* on the peritumoral lymphocytic reaction in CRC. It is conceivable that peritumoral T cells are subjected to different microbial and metabolic cues compared to those located intratumorally, potentially resulting in divergent immune phenotypes. Furthermore, the capacity of *F. nucleatum* to impair chemokine-mediated T cell trafficking suggests that it may contribute to the exclusion of effector lymphocytes from the tumor site, a hallmark of immune evasion. Given the prognostic significance of peritumoral lymphocytic reactions in CRC, future work should aim to dissect how *F. nucleatum* modulates the recruitment, activation, and spatial organization of T cells at the tumor periphery, which could represent another mechanism of immune evasion.

### Alteration of F.nucleatum on myeloid cells in the TME

Emerging evidence suggests that *F. nucleatum* also exerts profound effects on myeloid-derived immune cells within the TME, including macrophages, neutrophils, and myeloid-derived suppressor cells (MDSCs). These cells play pivotal roles in shaping tumor progression through modulation of inflammation, angiogenesis, and immune suppression.

#### F.nucleatum modulates macrophage polarization and secretion

**Figure 2.**
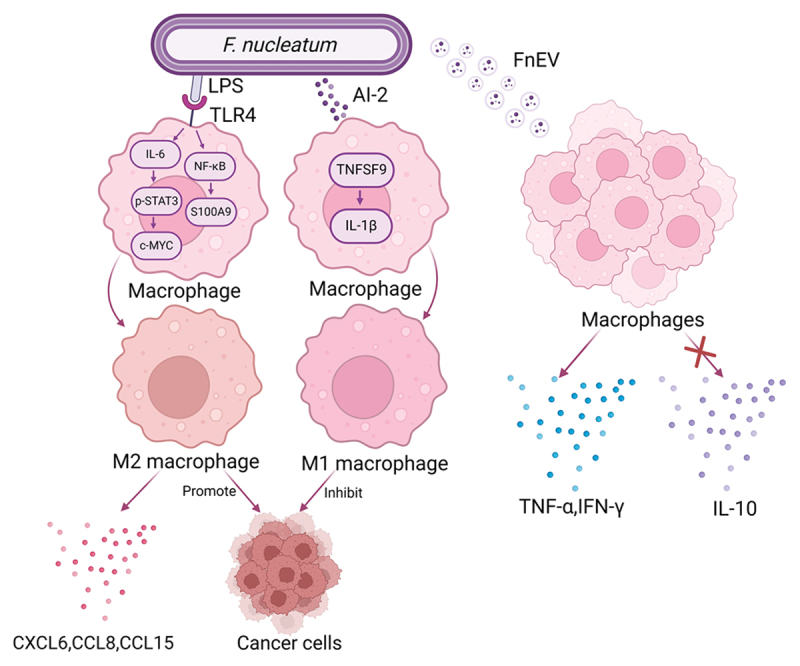
*F. nucleatum* regulates macrophage polarization and triggers inflammatory cytokine secretion. Lipopolysaccharides (LPS) of *F. nucleatum* can latches onto TLR4 on macrophages and initiate IL-6/p-STAT3/c-MYC and NF-κB/S100A9 axis, causing M2 polarization of macrophages and inducing inflammatory chemokines. Autoinducer-2 (AI-2) from *F. nucleatum* can engender M1 polarization through TNFSF9/IL-1β pathway. *F. nucleatum* extracellular vesicles (FnEvs) enhance inflammatory cytokine secretion and restrain anti-inflammatory cytokine production.

As shown in Figure 2, *F. nucleatum* regulates macrophage polarization and induces the release of pro-inflammatory cytokines that shape the TiME. Tumor-associated macrophages (TAMs) are predominantly composed of M2 macrophages, which facilitate tumor progression [62,63]. *F. nucleatum* has been observed to enrich TAM and M2 macrophages in the TME [64], potentially by inducing macrophage M2 polarization, thereby advancing CRC growth and metastasis [65–67]. Possible mechanisms involve elevating intracellular S100A9 via the TLR4/NF-κB pathway in both tumor cells and macrophages [65], as well as initiating the IL-6/p-STAT3/c-MYC pathway through the TLR4 receptor on macrophages [67]. Conversely, autoinducer-2 (AI-2) from *F. nucleatum* cause M1 polarization of macrophages via the TNFSF9/IL-1β pathway, converting them into the anti-tumor M1 phenotype, which makes AI-2 a potential therapeutic agent for CRC [68,69]. However, several questions remain unanswered regarding the specific molecular crosstalk between *F. nucleatum* and macrophage subtypes. It is unknown whether host factors such as iron status, which modulates macrophage polarization [70], may affect the outcome of *F. nucleatum*-macrophage interactions. Also, since AI-2 is an interspecies signaling molecule [71], it remains to be determined whether changes in microbial community composition can affect the M1/M2 macrophage balance by altering the production of AI-2 by *F. nucleatum*.

*F. nucleatum* can enhance macrophage secretion of inflammatory cytokines, forming a pro-inflammatory TME that supports tumor growth, invasion, and metastasis. FnEVs stimulate macrophages to secrete TNF-α and IFN-γ while inhibiting anti-inflammatory IL-10 [72]. LPS of *F. nucleatum* binds to TLR4 receptors on macrophages, activating NF-κB and inducing the secretion of inflammatory chemokines such as CXCL6, CCL8, and CCL15. Iron deficiency can impede protein phosphatase activity, inducing NF-κB p65 phosphorylation and subsequently suppressing *F. nucleatum*-induced macrophage secretion [73]. Moreover, *F. nucleatum* can trigger pyroptosis of macrophages to exacerbate tropical inflammation. LPS of *F. nucleatum* is capable of turning on caspase-11 in macrophages, subsequently initiating the cleavage of Gasdermin-D and triggering pyroptosis [74]. On top of that, *F. nucleatum* is capable of enhancing its own survival within macrophages. DNA hunger/stationary phase protective proteins (Dps) of *F. nucleatum*, which are ferritins protecting DNA from oxidative stress, can promote *F. nucleatum* survival within macrophages by the upregulation of chemokines CCL2 and CCL7 [75].

#### Effects of F.nucleatum on neutrophils

*F. nucleatum* significantly increases the number of tumor-associated neutrophils (TANs) in the TME [64]. TANs can be divided into N1 and N2 phenotypes, anti-tumor and pro-tumor, respectively. The plasticity of these two phenotypes are influenced by TME factors such as TGF-β, which induces N2 polarization [76], and low-dose type I IFN, which induces N1 polarization [77]. Therefore, TANs are likely to either promote or impede CRC progression depending on TME factors [78,79]. The dual effects of TANs on CRC progression indicate that studying the overall effects of *F. nucleatum* on TANs is insufficient, and further research should focus on the alteration of *F. nucleatum* on the two TAN phenotypes and their functional roles in CRC.

*F. nucleatum* facilitates CRC progression by stimulating the formation of neutrophil extracellular traps (NETs). NETs are extracellular web-like assemblies formed from certain proteins by which neutrophils capture and eliminate pathogens [80]. *F. nucleatum* stimulates NETs formation by initiating the TLR4-ROS signaling pathway and NOD1/2 receptors on neutrophils. NETs can promote CRC cell proliferation through angiogenesis, facilitate CRC cell migration, invasion, and adhesion by promoting epithelial–mesenchymal transition, and enhance the expression of invasion-related proteins. Circulating NETs may serve as a biomarker for forecasting CRC metastasis [81]. *F. nucleatum* also enhances angiogenesis in the TME by promoting neutrophil secretion of CXCL2 [82].

#### F.nucleatum recruits MDSCs to the TME

MDSCs are immunosuppressive cells that protect tumors from immune attack of the host immune system [83]. MDSCs are composed of two subsets: polymorphonuclear MDSCs (PMN-MDSCs), also termed as granulocytic MDSCs (G-MDSCs), and monocytic MDSCs (M-MDSCs), which resemble neutrophils and monocytes individually [84]. Studies have shown that MDSCs can aid CRC advancement through ROS and NO-mediated DNA damage [85] and immune suppression, bringing about poor CRC prognosis [86–89]. *F. nucleatum* can facilitate CRC advancement by recruiting MDSCs. In *F. nucleatum*-fed mice, both MDSC subsets, M-MDSCs and G-MDSCs, with their T cell suppressive activity, are considerably increased in intestinal tumors [64]. *F. nucleatum* also elevates MDSC density in CRC liver metastasis tissues [53]. Moreover, *F. nucleatum* can promote recruitment of PMN-MDSC to the TME via iNKT cells, which are gut effector T cells enriched in CRC lesions. *F. nucleatum* enhances iNKT cell expression of granulocyte-macrophage colony-stimulating (GM-CSF), facilitating PMN-MDSC recruitment [90]. Huang et al. found that S100A9 in the TME stimulates MDSC chemotaxis and activation [91]. Hu et al. discovered that *F. nucleatum* can enhance S100A9 expression in tumor cells and TME macrophages [65]. These two studies suggest that *F. nucleatum* may promote MDSC chemotaxis and activation through S100A9. Restraining *F. nucleatum*-mediated MDSC recruitment can delay CRC progression and improve prognosis. Dong et al. identified an M13 phage specifically binding to *F. nucleatum*. With silver nanoparticles assembled on its capsid proteins, it can eliminate *F. nucleatum*, reduce MDSCs in the TME, and, when combined with other normal treatments, extend the overall mouse survival in the orthotopic CRC model [92].

## The dual role of *F. nucleatum* in immune checkpoint blockade (ICB) therapy

Immune checkpoints hamper anti-tumor immune responses, and the use of antibodies to block cytotoxic T-lymphocyte-associated protein 4 (CTLA-4) or programmed death 1 (PD-1) pathways – achieving immune checkpoint blockade (ICB) – can enhance anti-tumor immune responses. This approach has shown substantial efficacy in various cancers [93,94]. The effectiveness of ICB therapy in CRC is closely related to the MSI status. As mentioned earlier, MSI status is directly associated with TMB and TIL density in CRC. Therefore, with lymphocyte infiltration and activity being major factors influencing ICB outcomes [95], the MSI status determines the efficacy of ICB therapy. MSI-H CRC, with its high TMB, high neoantigen load, and high TIL density, responds effectively to ICB [96]. Pembrolizumab and nivolumab, two humanized monoclonal PD-1 inhibitors, have been approved by the FDA for dMMR metastatic CRC, whereas pMMR CRC, due to lack of TIL, responds poorly to ICB [97]. For this reason, some perspectives suggest that dMMR CRC is hot tumor responsive to ICB, while pMMR CRC is cold tumor resistant to ICB. Combining ICB with treatments such as regorafenib may convert pMMR CRC into hot tumors, representing a promising approach for improving therapy outcomes [98,99].

**Figure 3.**
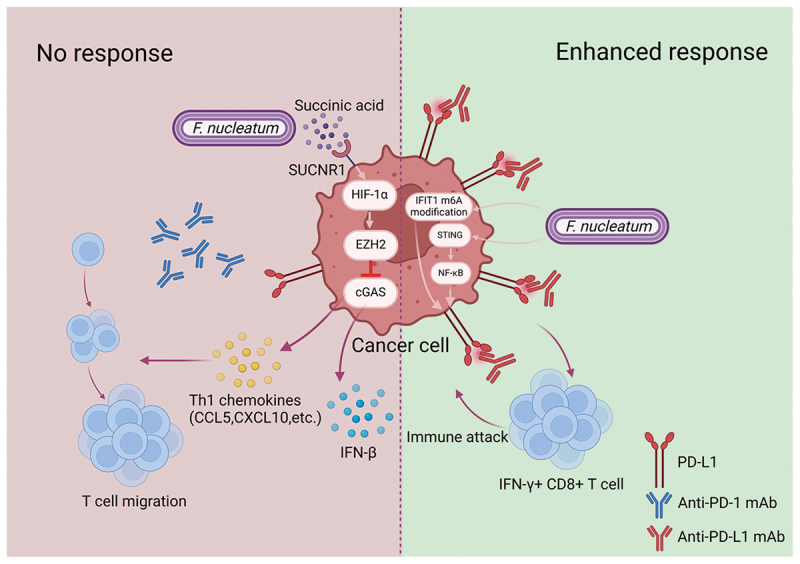
Double effects of *F. nucleatum* on immune checkpoint blockade (ICB). Succinate produced by *F. nucleatum* can activate the SUCNR1/HIF-1α/EZH2 axis and inhibit cGAS-mediated IFN-β and Th1 chemokine (such as CCL5 and CXCL10) production, thus suppressing the migration of CD8^+^ T cells to TiME, which eventually causes resistance to ICB. Upregulation of PD-L1 on cancer cells by *F. nucleatum* via IFIT1 m6A modification and STING/NF-κB activation can improve ICB efficacy. Immune attack is enhanced since *F. nucleatum* expands IFN-γ^+^ CD8^+^ TILs when PD-L1 inhibitors are administered.

As shown in Figure 3, current research results on how *F. nucleatum* influences ICB therapy responses are conflicting. *F. nucleatum* seems to have dual effects on ICB in CRC, possibly resisting or enhancing the efficacy through different mechanisms. As mentioned previously, Jiang et al. found that succinate, a metabolite of *F. nucleatum*, inhibits CD8^+^ T cell migration to tumor tissues, reducing TIL density, rendering anti-PD-1 antibody ICB therapy ineffective in mCRC [19]. Developing drugs targeting *F. nucleatum* as immunotherapy adjuvants can improve the efficacy of immunotherapy. Chen et al. developed a drug mimicking *F. nucleatum* by fusing *F. nucleatum* cell membrane with liposomes. This drug utilizes binding of Fap-2 on *F. nucleatum* to Gal-GalNAc over-expressed on the membrane of CRC cells to deliver antibiotics to CRC tissues overexpressing Gal-GalNAc. By eliminating *F. nucleatum*, this approach attenuates immunosuppression in the TME, and improves the efficacy of CTLA-4 and anti-PD-1 therapies [100]. These studies emphasize the negative impact of *F. nucleatum* on ICB therapy efficacy. However, others found contrasting results, Gao Yaqi et al. discovered that *F. nucleatum* induces programmed death ligand 1 (PD-L1) expression through m6A modification of IFIT1 [101]. Gao Yaohui et al. found that *F. nucleatum* stimulates PD-L1 expression by engaging the STING pathway. Moreover, with PD-L1 inhibitors applied, *F. nucleatum* increases IFN-γ ^+^ CD8 ^+^ TIL, thereby enhancing CRC response to PD-L1 blockade therapy [102]. Therefore, it is noteworthy that while *F. nucleatum*-induced upregulation of PD-L1 in tumor cells enhances immune evasion, concurrently upregulating PD-L1 expression during immunotherapy can convert cold tumors into hot tumors, improving immune therapy responses [103]. This reveals a beneficial role for *F. nucleatum* in enhancing the effectiveness of ICB therapy in CRC. Besides, in ESCC, *F. nucleatum*-Dps can enter ESCC cells, bind to the ATF3 site on the PD-L1 gene promoter, upregulating its expression [60], which is instructive for studying the mechanism of *F. nucleatum*-regulated PD-L1 expression in CRC.

There are several possible reasons for these seemingly contradictory results regarding the impact of *F. nucleatum* on ICB efficacy, especially the studies of Jiang et al. and Gao yaohui et al. In the analysis of clinical samples, Jiang et al. found that *F. nucleatum* attenuates the efficacy of immunotherapy in mCRC patients treated with PD-1 blockade and regorafenib therapy [19]. In contrast, Gao yaohui et al. revealed that *F. nucleatum* augments the efficacy of immunotherapy in CRC patients receiving PD-1 blockade [102]. The former study focused on mCRC patients, while the latter included both mCRC and non-mCRC ones. About 15% of CRC cases are MSI-H [52], whereas about 5% of mCRC cases are MSI-H [104]. As emphasized earlier, MSI status is closely related to immunotherapy efficacy and TIL density. Thus, the differing proportions of MSI-H CRC in mCRC and non-mCRC samples may lead to variations in TIL density, and the impact of *F. nucleatum* on TIL density also might differ between MSI-H and non-MSI-H CRC [45], potentially contributing to the observed differences in ICB efficacy between mCRC and non-mCRC cases. Also, given that the TiME evolves during CRC progression, the disease stage itself may influence how *F. nucleatum* affects immune dynamics and response to ICB therapy. Additionally, CRC metastasis is regulated by various immune cells within the TiME [105], including T-cells, TAMs, and MDSCs, implying that the TiME composition and characteristics differ between mCRC and non-mCRC, which may also result in differential alteration of *F. nucleatum* on ICB therapy response. Also, the influence of other drugs in combination with ICB therapy, such as regorafenib, cannot be ruled out. Furthermore, in in vitro and in vivo experiments, Jiang et al. used anti-PD-1 mAb, whereas Gao Yaohui et al. used anti-PD-L1 mAb, and this difference in ICB drug types may also account for the disparate results.

## Environment–host–microbe interaction in CRC: a molecular pathological epidemiology (MPE) perspective

Environmental and lifestyle factors have emerged as critical modulators of the gut microbiome, which in turn influences CRC development and progression. High-fat diet, low fiber intake, smoking, alcohol consumption and sedentary behavior have all been associated with gut dysbiosis or enrichment of oncogenic microbes, such as *F. nucleatum* [106–108]. These microbial effects are not uniform across individuals, as environmental exposures differ widely in frequency, intensity, and timing. Thus, understanding how lifestyle factors shape the *F. nucleatum*–host–tumor axis is essential to decipher the inter-individual variability in the TiME and clinical outcomes in CRC.

To address this complexity, integrative frameworks are needed to link external exposures with tumor molecular and microbial features. MPE is a transdisciplinary field that bridges epidemiology, molecular pathology, and microbiome science, enabling researchers to examine how environmental factors contribute to carcinogenesis through specific molecular alterations and microbial signatures [109–111]. In the context of CRC, *F. nucleatum* and TiME, MPE allows the integration of exposures, such as diet and smoking with microbial biomarkers like *F. nucleatum* abundance, alongside host molecular characteristics including MSI, and TiME patterns. By jointly analyzing microbial and molecular profiles within epidemiologic cohorts, MPE facilitates a deeper understanding of how modifiable risk factors interact with the TiME, leading to inter-individual differences in cancer progression and treatment response. For instance, an MPE study has demonstrated that diets with high intake of whole grains and fiber were associated with lower risk of *F. nucleatum*-positive CRC, but not *F. nucleatum*-negative tumors, suggesting a diet–microbe–tumor interaction specific to microbial subtypes [112]. Beyond this, by identifying microbial–molecular signatures that predict response to therapy or disease outcomes, MPE holds promise for contributing to precision prevention and microbiome-informed clinical strategies.

## Conclusion and future perspectives

The mechanisms by which *F. nucleatum* promotes CRC progression have become a well-established and rapidly evolving field of study. Extensive research indicates that *F. nucleatum* can interact with and influence various immune cells within the TiME, particularly impacting the infiltration of T lymphocytes and MDSCs, altering macrophage differentiation, and disrupting the normal functions of various immune cells. These interactions contribute to the establishment of an immunosuppressive TiME, ultimately promoting the progression of CRC. While *F. nucleatum* is generally considered to result in an immunosuppressive TME and facilitate tumor progression, under certain conditions, it might contribute to anti-tumor immunity instead. For instance, as previously mentioned, *F. nucleatum* might be positively related with TIL density in non-MSI-H CRC [45] and may contribute positively to PD-L1 blockade therapy [102]. Besides, *F. nucleatum*-secreted AI-2 induces M1 polarization of macrophages [69]. These exceptions highlight the complexity of interactions between *F. nucleatum* and TiME and suggest that its role in tumor immunity may be context-dependent.

Despite these findings, the current understanding of *F. nucleatum*–mediated immune modulation in CRC remains incomplete. One challenge lies in the heterogeneity of *F. nucleatum*. Recent studies have revealed considerable genetic and functional heterogeneity among *F. nucleatum* subspecies and clinical isolates [113–115]. These differences may influence the bacterium’s ability to modulate immune cell responses, alter cytokine production, and impact epithelial barrier integrity, possibly contributing to inconsistencies across experimental models and clinical observations. Future research should incorporate bacterial genomics, strain isolation, and functional assays.

In addition, although existing studies have investigated the impact of *F. nucleatum* on individual immune cell subsets, such as T cells, macrophages, and MDSC, the TiME operates as a dynamic and interconnected network. Currently, few studies have systematically mapped how *F. nucleatum* modulates the TiME at a system level. Nevertheless, scattered mechanistic evidence suggests that *F. nucleatum* may shape the immune landscape through indirect signaling and intercellular feedback. For instance, *F. nucleatum* has been shown to promote the expansion of MDSC, M2-like TAM and CD103^+^ dendritic cells. These immune cell populations suppress CD4^+^ T cell activity through iNOS, arginase-1, and regulatory T cell induction [64]. These findings underscore the need to more comprehensively map the network-level impact of *F. nucleatum* on the TiME.

Moreover, how *F. nucleatum* modulates the TiME across different stages of CRC progression remains unclear, as few studies have systematically compared its immunological effects across early, advanced, and metastatic CRC. Most experimental models represent a single disease stage, and human studies rarely stratify by tumor stage when analyzing *F. nucleatum*–TiME interactions. Future work should prioritize longitudinal, stage-resolved investigations to address this gap. Additionally, recent spatial transcriptomic studies in CRC have revealed region-specific immune cell niches across the TiME [116]. However, the spatial localization of *F. nucleatum* within the TiME remains largely uncharacterized. Given the compartmentalized nature of immune responses in CRC, *F. nucleatum* may exert context-dependent effects depending on its spatial proximity to specific immune cell populations.

Beyond mechanistic insights, several *F. nucleatum*-related directions have emerged as promising areas for therapeutic potential, including targeting and biomarker development. Given that *F. nucleatum* promotes CRC development not only through TiME regulation but also through various other mechanisms, targeting *F. nucleatum* appears promising as an adjunctive treatment to conventional therapies. Recent preclinical studies have demonstrated the efficacy of various strategies to reduce *F. nucleatum* burden, including antibiotics, nanomaterials, and engineered vaccines [19,100,117,118]. For example, a cholesterol-modified CpG oligonucleotide (Chol-CpG) – loaded bacterial membrane vaccine has shown the ability to selectively eliminate *F. nucleatum*, thereby mitigating chemoresistance and metastasis [119]. Tellurium- and cisplatin-loaded nanocarriers exhibit combined antibacterial and chemotherapeutic effects [120], while targeted photothermal systems conjugated with GalNAc ligands offer *F. nucleatum*–specific ablation under near-infrared activation [121]. Additionally, bacteriophage-based approaches have shown specificity in targeting *F. nucleatum* [122,123]. Concurrently, the potential of *F. nucleatum* as a biomarker for prognostic evaluation and ICB therapy assessment also demands follow-up studies. Its abundance in tumor tissue and fecal samples may correlate with immune infiltration patterns and responses to ICB in certain cohorts. Future work should focus on standardization of detection methods and validation in clinical cohorts.

## Additional information

Correspondence and requests for materials should be addressed to Xin Zheng.
